# Do Fleas Affect Energy Expenditure of Their Free-Living Hosts?

**DOI:** 10.1371/journal.pone.0013686

**Published:** 2010-10-27

**Authors:** Michael Kam, A. Allan Degen, Irina S. Khokhlova, Boris R. Krasnov, Eli Geffen

**Affiliations:** 1 Desert Animal Adaptations and Husbandry, Wyler Department of Dryland Agriculture, The Jacob Blaustein Institutes for Desert Research, Ben Gurion University of the Negev, Beer Sheva, Israel; 2 Mitrani Department of Desert Ecology, The Jacob Blaustein Institutes for Desert Research, Ben Gurion University of the Negev, Sede Boqer Campus, Midreshet Ben Gurion, Israel; 3 Department of Zoology, Tel Aviv University, Tel Aviv, Israel; Universidad Europea de Madrid, Spain

## Abstract

**Background:**

Parasites can cause energetically costly behavioural and immunological responses which potentially can reduce host fitness. However, although most laboratory studies indicate that the metabolic rate of the host increases with parasite infestation, this has never been shown in free-living host populations. In fact, studies thus far have shown no effect of parasitism on field metabolic rate (FMR).

**Methodology and Results:**

We tested the effect of parasites on the energy expenditure of a host by measuring FMR using doubly-labelled water in free-living Baluchistan gerbils (*Gerbillus nanus*) infested by naturally occurring fleas during winter, spring and summer. We showed for the first time that FMR of free-living *G. nanus* was significantly and positively correlated with parasite load in spring when parasite load was highest; this relationship approached significance in summer when parasite load was lowest but was insignificant in winter. Among seasons, winter FMRs were highest and summer FMRs were lowest in *G. nanus*.

**Discussion:**

The lack of parasite effect on FMR in winter could be related to the fact that FMR rates were highest among seasons. In this season, thermoregulatory costs are high which may indicate that less energy could be allocated to defend against parasites or to compensate for other costly activities. The question about the cost of parasitism in nature is now one of the major themes in ecological physiology. Our study supports the hypothesis that parasites can elevate FMR of their hosts, at least under certain conditions. However, the effect is complex and factors such as season and parasite load are involved.

## Introduction

Parasites derive their food and other biological supplies from their hosts [1], [2]. They may affect the host directly, by reducing the resources of the host, and indirectly, by causing energy costly behavioural [3]–[6] and/or immunological [7], [8] responses. Although parasite infestation can cause the host to increase food intake [9], most studies have shown that parasites suppress food consumption of their hosts [10], [11], [12], which could reduce the energy allocation for production and, consequently, reduce host fitness. Generally, ectoparasites are small in comparison with their hosts and the energy content of the resources they consume from their hosts is a very small proportion of the hosts' energy expenditure [13]. Consequently, the direct effects on energy balance of the host are negligible [14], as would be the case with fleas and their hosts. Nonetheless, fleas, even in small numbers, can cause a significant increase in energy requirements of the host (indirect effect of parasite) [15], [16].

Some laboratory studies have demonstrated detrimental effects of parasites on their hosts [17], [18], while others have failed to do so [19]. Furthermore, some studies reported an increase in basal metabolic rate [20], resting metabolic rate [5], [21], [22] and maintenance energy requirements (average daily metabolic rate, ADMR; [15], [23]) of parasitized compared to non-parasitized individuals. However, the evidence of the energetic effect of fleas on the host is equivocal as, at least in one case (*Gerbillus andersoni* infested with the flea *Synosternus cleopatrae*), parasitized hosts did not increase ADMR above that of non-parasitized hosts [23] and in another case, (*Xerus inauris* which was treated to remove endo- and ectoparasites) parasitized hosts had a resting metabolic rate lower than that of parasite-free hosts [24]. Physiological defense strategies of hosts against parasites provide a possible explanation for these different energetic responses of infested individuals.

Two main physiological responses of the host to parasite infestation have been reported. Firstly, there is an increased metabolic rate due to the energy costs of activating and maintaining the immune system, as was suggested for the increased metabolic rate in both avian [21] and mammalian [23], [25] hosts. Secondly, the response of the host depends on the level of immunological “readiness” to flea infestation. Hosts possessing an induced immune response to fleas elevate their energy metabolism when parasitized whereas hosts possessing a constitutive immune response maintain a constantly high level of energy metabolism to combat parasites [23], [26].

Yet, in spite of the variety of responses to parasite infestation, most laboratory studies indicate that the metabolic rate of the host increases with parasite infestation. Surprisingly, this occurrence has never been shown in free-living host populations although the question concerning the cost of parasitism in nature has drawn much attention recently in ecological physiology. In fact, studies thus far have shown no effect of parasitism on field metabolic rate (FMR) [24], [27].

We hypothesized that parasites affect the energy expenditure of their hosts and predicted that FMR of free-living hosts would increase with parasite load. To test our prediction we used rodents and fleas, one of the most common host-parasite systems among mammals [13], [28], [29]. We simultaneously measured FMR and ectoparasite load in free-living Baluchistan gerbils, *Gerbillus nanus*, a granivorous desert rodent. We reasoned that other factors known to affect energy expenditure interplay and, thus, included a large sample size that covered three seasons and both sexes, which permitted inter- and intra-seasonal analyses. We report, for the first time, the increased energy expenditure of a free-living rodent due to ectoparasites.

## Methods

### Ethics Statement

This study was conducted under permit number 1998/3853 from the Israel Nature and National Parks Protection Authority and satisfying the requirements of the Ben-Gurion University Committee for the Ethical Care and Use of Animals in Experiments.

### Study Site and Animals

The study was conducted in the Sheizaf Nature Reserve located in the Rift Valley (30°45′N, 35°15′E), about 30 km south of the Dead Sea, Israel. This site is in the northern part of the Arabian Sahara Desert and is characterized by long, hot, dry summers. It is extremely arid [30] with a mean annual winter rainfall of 35 mm that falls in an average of 9·6 days, but there are large annual variations in total rainfall and in its temporal and spatial distribution. Evaporation averages 3400 mm annually and in summer averages 14 mm daily. The total annual dew is less than 1 mm. Average daily air temperature for the hottest (July– August) and coldest (January–February) months are 30°C and 15°C, respectively. Average maximum daily air temperature for July–August is 38°C, with temperatures reaching 47°C, and average minimum daily air temperature for January–February is 9°C, rarely dropping below 0°C [31]. The soil is of the Hatzeva formation which is known for its ability to hold water and, as a result, the area has more vegetation than other deserts of similar rainfall.

Minimum and maximum air temperatures during our study averaged 11·0±2·2°C and 20·7±1·9°C, respectively, in winter (December), 15·9±2·1°C and 28·6±4·1°C, respectively, in spring (March), and 24·1±1·7°C and 38·1±2·8°C, respectively, in summer (June) and total precipitation was 22·8 mm (Yair Station, Arava R&D, Israel).

We set 300 Sherman traps in winter (December), spring (March) and summer (June), to capture Baluchistan gerbils, (*Gerbillus nanus* Blanford, 1975). The traps, baited with millet seeds, were set after sunset and were checked for gerbils early morning at first light. *Gerbillus nanus* is a nocturnal, granivorous desert rodent and, as other *Gerbillus* species, is capable of maintaining energy and water balances over prolonged periods on millet seeds. All fleas were removed manually over a large tub, counted and identified [32]; no fleas were found off the host and free in the traps. The number of fleas collected from the host was assumed to be a constant proportion of the flea reservoir within rodent burrows, as was reported for other rodents including gerbils of the same genus, *Gerbillus* [16], [33]. In this study, the number of fleas collected on re-capture (2.3±2.8) was not lower than that of first capture (1.6±2.1); consequently, flea removal did not decrease the number of fleas during FMR measurements (see below). Flea load was taken as the average of the two measurements, that is, at capture and re-capture. We identified three species of fleas (*Xenopsylla conformis*, *Nosopsyllus pumilionis* and *Parapulex chephrenis*), of which *Xenopsylla conformis* was the most common. *P. chephrenis* was rarely found on *G. nanus*; it is a specific flea on the spiny mouse, *Acomys cahirinus* that occurs on a nearby habitat, and its presence on *G. nanus* could be a result of encounters between the two rodent species [16].

### Field Metabolic Rate Measurements

Field metabolic rate of non-reproducing, mature, free-living *G. nanus* was measured using the doubly-labelled water method (DLW, ^3^HH^18^O [34]–[36]. Captured individuals were carefully checked for fleas, weighed to 0.01 g and injected intraperitoneally with 0.150 ml of DLW containing 97 atoms percent ^18^O (Rotem Industries, Beer Sheva, Israel) and 100 µCi tritium per ml (New England Nuclear, Boston, MA). Two hours were allowed for equilibration of the isotopes with body fluids [37], during which time food was unavailable, and then a blood sample (about 60 µl) was collected from the infraorbital sinus using a haematocrit capillary tube (38). The animals were then weighed and released at the point of capture. Animals were recaptured 2 – 5 days later, checked for fleas, and a second blood sample was collected. They were then weighed and released.

Blood samples were micro distilled [38] and analyzed for ^18^O concentration and ^3^H levels (at the Boston University Stable Isotope Laboratory). Total body water volume was estimated from the initial dilution of ^18^O, calculation of CO_2_ production followed equation 2 of Nagy [35] and 21.9 J/ml CO_2_, suitable for granivorous rodents [38], [37], was used to calculate energy expenditure. During measurements of FMR, body mass of the gerbils did not differ between capture and re-capture and the measuring periods over which blood samples were taken consisted of ∼24 h periods (24.17 ± 0.15 h; n = 105), thus satisfying the requirements of the doubly-labelled water method [35], [36].

### Data Analyses

In total, FMR was measured in 105 individual *G. nanus*, consisting of 29 females and 76 males. We defined several variables including presence of fleas, that is, animal with or without fleas, parasite prevalence in the population, number of fleas, and season (winter, spring and summer taken as the average temperature during the activity period of the rodents). A linear model was used to examine the effect of the number of fleas on *G. nanus* FMR by season, and included sex and body mass as covariates. Body mass, sex and number of fleas were added sequentially to the model such that body mass was added first and number of fleas last. This sequential procedure allowed us to evaluate the net contribution of the effect of fleas to the regression model. Statistical tests were made using SPSS (version 17, SPSS Inc.). We also presented FMR values per body mass to the power of 0.622, which is used for comparison of FMR among different body-sized rodents [39]. Values are presented as means ± SD.

## Results

Body mass of *G. nanus* was lower in winter (20.2 ± 2.4 g; n = 43, F_2,102_ = 14.9, P<0.0001) than in spring (22.8 ± 2.2, n = 37) and summer (22.7 ± 2.5, n = 25) and differed between sexes (20.7 ± 2.5 g, n =  29 and 22.1 ± 2.6 g, n = 76 in females and males, respectively, F_1,103_ = 8.2, P = 0.005). The interaction between season and sex was insignificant (F_2,102_ = 2.2, P = 0.121). Total body water (68.3 ± 2.6% of body mass), controlled for body mass, was similar between sexes and among seasons (season: F_2,102_ = 2.7, P = 0.074; sex: F_1,103_ = 0.9, P = 0.349; sex*season: F_2,102_ = 0.6, P = 0.566).

**Figure 1:**
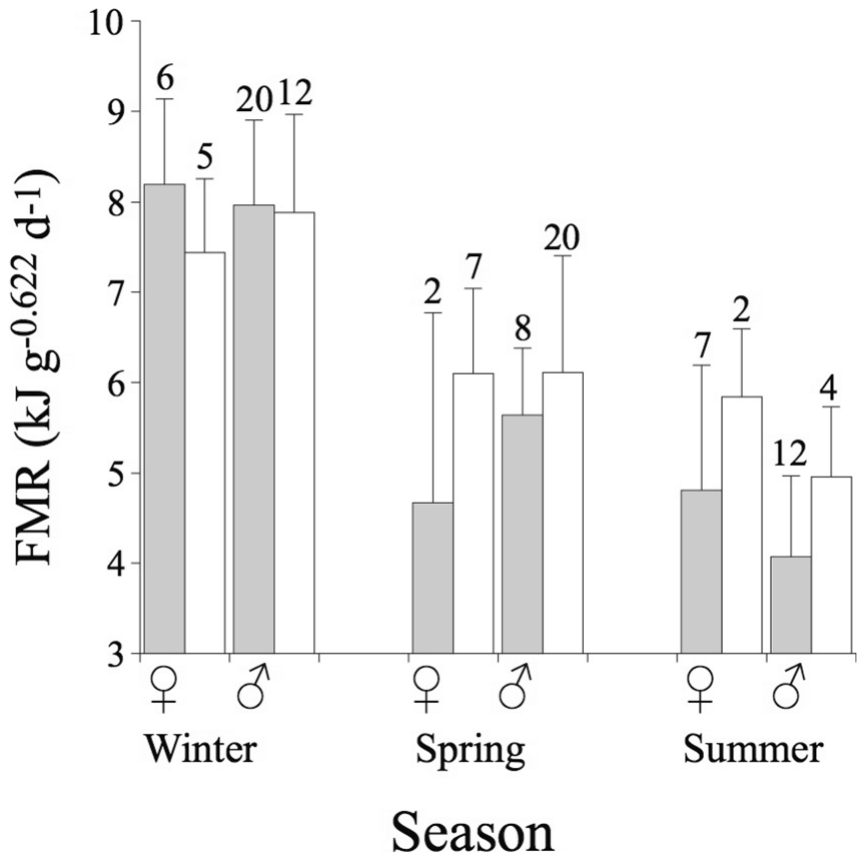
Average field metabolic rates (FMR) in parasitized (empty bars) and non-parasitized (filled bars) *Gerbillus nanus* in different seasons. Average FMRs (± S.D.) of males (♂) and females (♀) are presented per metabolic mass [37] and numbers above error bars denote sample sizes.

Within each season, FMR of *G. nanus* was similar in males and females (F_1,103_ = 0.2, P = 0.641; season*sex: F_2,102_ = 2.5, P = 0.085). However, FMRs, both absolute (kJ d^−1^; F_2,102_ = 33.8, P<0.0001, Fig. 1) and mass specific (F_2,102_ = 48.4, P<0.0001), were significantly different among seasons being highest in winter (51.2 ± 7.3 kJ d^−1^, 7.91 ± 0.96 kJ g^−0.622^ d^−1^, n = 43), lowest in summer (31.8 ± 8.5 kJ d^−1^, 4.56 ± 1.12 kJ g^−0.622^ d^−1^, n = 25) and intermediate in spring (41.4 ± 8.9 kJ d^−1^, 5.93 ± 1.18 kJ g^−0.622^ d^−1^, n = 37).

Flea load ranged from zero to eight in winter, zero to nine in spring and zero to three in summer with respective means of 0.81 ± 1.56, 2.25 ± 2.51 and 0.48 ± 0.92 fleas per individual. Both males and females had significantly more fleas in spring than the other seasons (one-way ANOVA by randomization; F_2,26_ = 5.3, P = 0.006 and F_2,72_ = 4.3, P = 0.018 for males and females, respectively). The number of fleas collected off males and females was not significantly different (randomization test; P = 0.412).

The number of fleas had a significant effect on FMR in *G. nanus* in spring and approached significance in summer (Table 1); flea load explained 13% and 21% of the variance in FMR, respectively. Sex did not have a significant effect on FMR in any season (Table 1). The interaction sex*fleas was also insignificant in all seasons, and thus was not included in the analyses.

**Table 1:** The effect of number of fleas (Fleas) on FMR of *Gerbillus nanus* by season and sex. Body mass (m_b_) and sex were added first to the model as covariates.

Season	Effect	F	*df*	*P*	r^2^	Power
Winter	Model	4.8	3,39	0.006	0.27	
	m_b_	13.7	1,41	0.001	0.25	
	Sex	0.1	1,40	0.784	0.00	0.059
	Fleas	1.1	1,39	0.304	0.02	0.174
Spring	Model	4.2	3,32	0.013	0.28	
	m_b_	6.2	1,34	0.018	0.15	
	Sex	0.9	1,33	0.877	0.00	0.057
	Fleas	5.7	1,32	0.023	0.13	0.636
Summer	Model	4.0	3,21	0.021	0.36	
	m_b_	4.4	1,23	0.048	0.16	
	Sex	2.4	1,22	0.138	0.08	0.373
	Fleas	4.0	1,21	0.058	0.12	0.483

Although the overall sample size of 105 FMR measurements is one of the largest for similar field studies, the biased sex ratio in each season resulted in smaller sample sizes for females than males. Could this affect the detection of differences in FMR between sexes? Power analysis (Table 1), showed low values for spring and winter samples, thus sample size for females may be insufficient. Nevertheless, the amount of variance accounted by sex during spring and winter approached zero, suggesting that sex did not affect FMR in these seasons.

## Discussion

### Energetics of Free-Ranging Individuals

Field metabolic rate (FMR) is a measure of energy expenditure of an animal under natural free-living conditions [39]. Several studies on the effect of parasites on host energetics were made in free-ranging species, but energy expenditure in these hosts was measured in the laboratory as basal metabolic rate [20] or resting metabolic rate [21], [22]. FMR includes basal and resting metabolic rates, however, other expenses such as the heat increment of feeding, thermoregulation, predator avoidance and foraging are not included in laboratory measurements. These energy costs are significant [40] as FMR in small avian or mammalian species could be greater than four times BMR [41]. Therefore, effects of parasites on the energy expenditure of free-ranging hosts cannot be concluded reliably from studies measuring metabolic rate in the laboratory. Effects on host metabolic rate under laboratory conditions could be useful for raising testable hypotheses on the energy expenditure of free-ranging hosts, which is the basis for the present study.

### Parasitism and Field Metabolic Rate of Hosts

To date, two studies have tested the effect of parasitism on FMR, with both reporting no effect. Doubly-labelled water measurements over one day in 15-day old house martin nestlings showed that daily energy expenditure did not differ between infested hosts and controls. But most individuals in all treatments, including controls, lost body mass during the measuring period [27], suggesting that the experimental procedure affected the well being of focal individuals. Also, changes in body mass over the measuring period could lead to errors in FMR estimates [35], [36]. In another study, FMR did not differ between parasitized and non-parasitized (parasites removed) Cape ground squirrels. However, as noted by the authors, the measurements of the two groups were not done concurrently and, consequently, there was no control for the effect of time [24].

In the present study, parasite load significantly affected FMR of *G. nanus* in spring, thus supporting our hypothesis. However, the effect of parasites on energy expenditure of the hosts was complex; it varied with season, approaching significance in summer, at which time parasite load was lowest and was not significant during winter, at which time FMR reached its peak. As in other small rodents, including gerbils [37], FMR was highest in winter. Possible reasons for this are high thermoregulatory responses and the long scotophase [42], [43]. Such diverse responses could be the reason why the other field studies [24], [27] failed to detect a difference in FMR between infested and parasite-free hosts. It should be noted that when no parasite effect on FMR is detected, it may not necessarily imply no immunological costs, as the response could be dependant on the immunological “readiness” of the host which could present a constitutive immune response. Such a constitutive immune response would be advantageous at high parasite load; results of this study may suggest that this is not the case with *G. nanus* as parasite load was not highest in winter.

When a host is infested by parasites, it is confronted with trade-off decisions between the energy costs of the immune defense system and other energy demanding processes [12] and could possibly compensate for energy expenses due to parasitism by reducing other activities such as social or predatory responses [44]. For example, blue tits with induced anti-body responses reduced their nestling feeding rates compared to controls [45]. Based on these reports, we reasoned that the lack of parasite effect on FMR of the host in winter was not a result of “no effect”, but rather reflected a decrease in energy allotment to other activities. As winter requires higher thermoregulatory costs and is also a critical season for mating and maintaining suitable territory to support reproduction, parasitized *G. nanus* may be more susceptible to predation and/or are less successful in mating during winter. We suggest this scenario as a possible mechanism through which parasites can affect the fitness of individuals.

### Consequences of the Experimental Design

In this study, the natural population of both the host and its parasites were not manipulated. Thus, the question of cause and effect arises, that is, whether the parasites affected FMR or the parasites chose individuals with high FMR. We concluded that the elevated FMR was due to parasite infestation and here we present rationale to support our conclusions. Adult fleas usually alternate between periods when they occur on the host body for feeding and periods when they occur in its burrow or nest. In most cases, pre-imago development is entirely off-host. They are rarely active in the host burrow or on the host body and are thus limited in their ability to choose a host. As a result, they infest mainly resident rather than dispersing host individuals [16], [46]. As *G. nanus* is a solitary gerbil, it is reasonable to assume that infestation of a host individual is not related to selection by fleas.

In multi-factorial designs, comprehensive analyses of all independent factors/variables (i.e., season, ambient temperature and flea load) to study the partial effect of each one on the dependant variable (i.e., FMR) are generally presented. However, in this study, the collinearity between the season and flea load would not allow for such an analysis. Therefore, FMR was analyzed separately within each season.

The question about the cost of parasitism in nature is now one of the major themes in ecological physiology. We presented a study employing a specific host-parasite system that demonstrated a variety of responses. Our study supports the hypothesis that parasites can elevate FMR of their hosts under certain conditions. However, the effect is complex and factors such as season and parasite load, among others, are involved.
